# Bilateral Necrotizing Scleritis with Scleral Melt associated with Herpes Simplex Infection: A Case Report

**DOI:** 10.22336/rjo.2023.64

**Published:** 2023

**Authors:** Arti Singh, Ashutosh Maharana, Srishti Nagarajan, Shubhi Sachan

**Affiliations:** * Department of Ophthalmology, Motilal Nehru Medical College, Prayagraj, Uttar Pradesh, India

**Keywords:** necrotizing scleritis, scleral melt, herpes simplex keratitis, bilateral

## Abstract

**Objective (Aim):** The article is a case report of a very rare case of bilateral herpes simplex virus infection associated with bilateral necrotizing scleritis with scleral melt in an elderly north Indian female of lower middle socioeconomic status.

**Methods:** A 65-year-old female presented to our clinic with a wide variety of presentations ranging initially from neurotropic corneal ulcer to necrotizing scleritis with scleral melt for 2 years. The patient records were evaluated and computed. A PubMed literature search on herpes scleritis was conducted and reviewed.

**Results:** A keen sense of judgment, timely management, and patient counseling are crucial for a rapid and favorable outcome.

**Conclusions:** Bilateral necrotizing scleritis with scleral melt can be a rare atypical presentation of herpes simplex keratitis. In such atypical cases, diagnosis may be challenging. Associated clinical findings, history of herpes keratitis, which may be recurrent, and response to antiviral drugs, may give clues towards the diagnosis in such atypical cases. In addition to this, surgical intervention should not be delayed if it seems inevitable.

**Abbreviations:** RE = right eye, LE = left eye, BCL = bandage contact lens, KP = keratic precipitate, mm = millimeter, mg = milligram

## Introduction

Inflammation of the sclera is termed Scleritis, which is a rare ocular inflammatory disease caused by occlusive vasculitis of the deep episcleral plexus with a risk of ischemia and necrosis [**1**]. It can be classified anatomically as anterior or posterior, depending on the site of the lesion. Anterior scleritis is further classified into diffuse, nodular, and necrotizing, depending on the type of lesion [**2**,**3**]. Immunological etiologies have been implicated in most of cases (90%-95%) of scleritis while 5%-10% of cases of scleritis develop from infections [**4**-**8**]. Although rare, an infectious etiology may be considered in cases of scleritis, especially those that are long-standing, fail to respond to standard therapies, or are of necrotizing type. 

Herpes keratitis has a global incidence of 1.5 million yearly with nearly 40,000 cases contributing to severe visual impairment, although its prevalence depends upon age, socioeconomic status, and geographic location [**9**,**10**]. 

Ophthalmic manifestation of herpes infections includes blepharitis, conjunctivitis, epithelial keratitis, stromal keratitis, iritis, cyclitis, posterior uveitis, and retinitis. Necrotizing scleritis is one of the ophthalmic manifestations of herpes infection that results in severe visual impairment. This association is highly uncommon and only a handful of cases have been reported [**11**-**16**].

This case report presents an unusual case of herpes infection associated with bilateral necrotizing scleritis with scleral melt in one eye in an elderly north Indian female of lower middle socioeconomic status.

## Case presentation

**Fig. 1:**
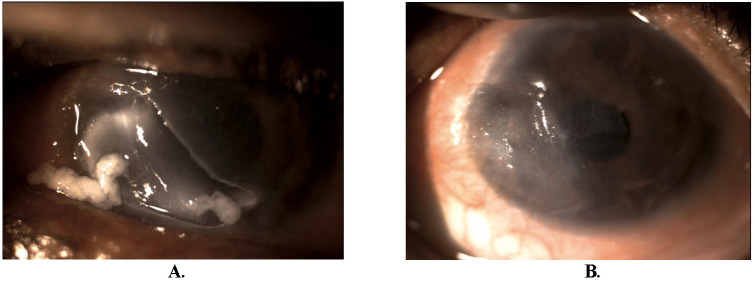
**A.** Initial presentation with large ulcer having a smooth base and margins extending from 10 to 5 o’clock mid-peripheral to peripheral cornea associated with peripheral corneal thinning. **B.** Healed corneal ulcer with thinning of the peripheral cornea

We report a case of a 65-year-old, north Indian female of lower middle socioeconomic status from a rural community in Prayagraj, Uttar Pradesh, India, who presented in our clinic around 2 years back with a history of pain, redness, photophobia, and diminution of vision in the right eye (RE). She had a history of uneventful cataract surgery in both eyes 5-6 years back. She also had similar complaints in her left eye (LE) a few years back of which no documents were available. According to the patient, no systemic co-morbidity existed. Her vision was finger counting in RE and 6/36 in LE. On slit-lamp examination, a large corneal defect, having a smooth base and margins with no infiltrate, was observed, extending from 10 to 5 o’clock and was associated with peripheral corneal melting in RE (**Fig. 1**). Her Schirmer’s value was 10 mm in RE and 12 mm in LE. Corneal sensations were diminished in RE. Based on clinical presentation, she was diagnosed with neurotrophic corneal ulcer stage 3 with peripheral corneal melting (RE). She was started on topical lubricating eye drops in RE and a bandage contact lens (BCL) was also applied. The defect healed in 6 weeks with peripheral corneal thinning when BCL was removed and lubricating drop was continued.

**Fig. 2:**
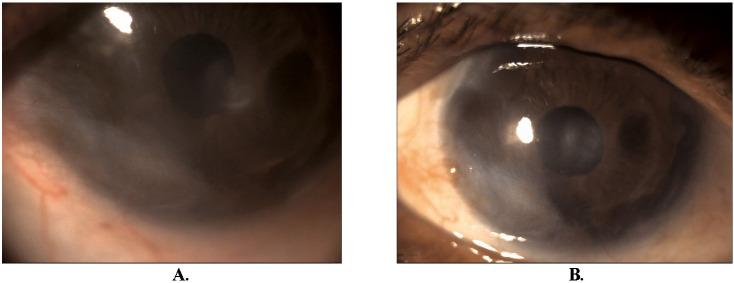
**A.** 2nd episode showing epithelial keratitis with a greyish elevated margin with subepithelial infiltrate in the central cornea along with a few medium-sized grey keratic precipitate (KP) with 2+ cells and mild flare. **B.** Presentation after 2 weeks showing healed corneal lesions and no KPs

The patient remained asymptomatic for 2 months when she presented again with pain, redness, and watering in the same eye (RE). Slit-lamp examination showed epithelial keratitis with a greyish elevated margin with subepithelial infiltrate in the central cornea along with a few medium-sized greyish keratic precipitates (KPs) with 2+ cells and mild flare (**Fig. 2**). Typical corneal lesion and anterior chamber reaction along with the previous clinical history of the patient, suggested the diagnosis of herpetic kerato-uveitis. Oral Acyclovir 400 mg 5 times a day for 14 days was administered, thereafter the dosage was reduced to twice a day. Along with systemic medications, topical corticosteroids, and lubricating eye drops were administered in RE. She showed improvement within 2 weeks and thus topical corticosteroids were tapered and she was kept on follow-up with lubricating eye drops and oral Acyclovir 400 mg twice a day. A detailed dilated fundus evaluation and refraction were also performed. Her fundus examination was within normal limits and visual acuity improved to 6/24 in RE and 6/12 in LE with spectacles. 

**Fig. 3:**
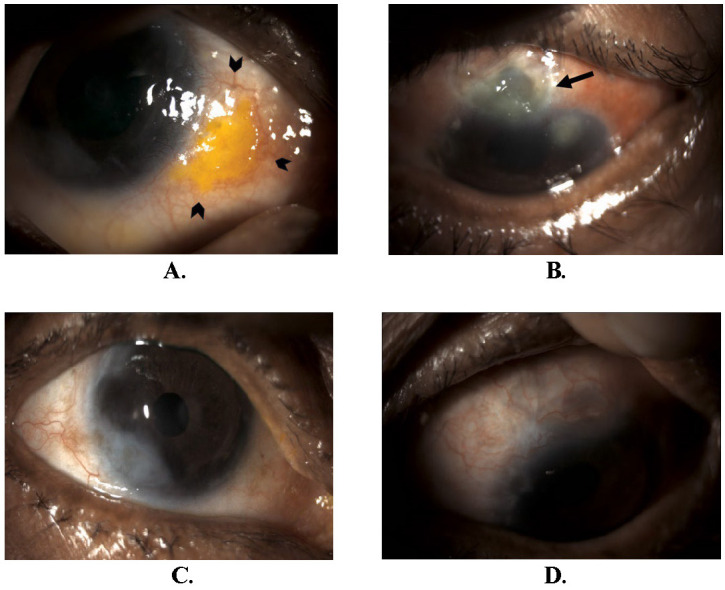
**A.** 3rd episode showing an inferotemporal scleral necrotic lesion (arrowheads) at 3-5 o’clock near limbus in RE. **B.** At the same visit, the patient also had scleral necrosis with scleral melt at 12 o’clock (arrow) and at 9-10 o’clock near limbus along with epithelial keratitis lesion at 1 o’clock in LE. **C.** Presentation after 3 weeks showing healed scleral lesion in RE. **D.**Presentation after 3 weeks showing healed scleral and corneal lesions in LE

She remained asymptomatic for around 1.5 years when she presented again with complaints of pain, redness, and watering, this time in both eyes. On slit-lamp examination, a scleral necrotic inferotemporal lesion at 3-5 o’clock near limbus in RE and scleral necrosis at 12 o’clock and at a 9-10 o’clock near limbus along with an epithelial keratitis lesion at 1 o’clock and hypopyon in LE were found (**Fig. 3**). Conjunctival scrapping was sent for gram staining and culture sensitivity, which came out to be negative for any microbial growth. Clinical findings and typical epithelial keratitis lesion suggested the recurrence of herpetic infection with necrotizing scleritis, which progressed to scleral melt in LE. A conjunctival advancement flap was performed to cover the scleral melt at 12 o’clock in LE under sterile aseptic conditions. Oral Acyclovir 400 mg was started 5 times a day along with topical antibiotic and lubricating eye drops in both eyes and in addition, topical corticosteroid eye drops were started in RE. She showed gradual improvement in signs and symptoms and scleral necrosis and corneal epithelial keratitis lesions gradually disappeared and only a few superficial punctate keratitis lesions were seen at the end of 3 weeks post-surgery.

## Discussion

Herpes keratitis is one of the leading causes of infectious keratitis worldwide and can involve any part of the eye. The manifestations range from dendritic ulcer, and necrotizing stromal keratitis, to scleritis, retinitis, and peripheral ulcerative keratitis. 

The prevalence of scleritis associated with herpes infection varies between 4.2% to 7.5% in different published literature [**5**,**17**,**18**]. Findings vary from diffuse scleritis, nodular scleritis, and necrotizing scleritis to scleromalacia. 

While in some cases the patient has a typical clinical appearance of dendritic ulcer and the pathological process is straightforward, there are many cases in which this demarcation seems blurry and diagnosis may be challenging. Scleritis is one such atypical presentation of herpes infection. Although herpetic scleritis is not uncommon, necrotizing scleritis type associated with herpes infection has been rarely seen and only a handful of cases have been reported. On thorough literature search, we obtained a total of 4 cases of necrotizing scleritis, most of them being unilateral and without any scleral melt [**11**,**12**,**14**]. Although, the diagnosis can be confirmed on conjunctival scleral biopsy, polymerase chain reaction analysis of the aqueous sample, and positive serum anti-herpes virus titers, in most cases the decision has to be made on clinical grounds [**11**-**16**]. The literature search performed on herpetic scleritis was tabulated (**Table 1**).

**Table 1:** Summary of the literature search on herpetic scleritis

Year and Journal	Author	Number of cases	Types of scleritis	Diagnosis	Treatment
2003 *Eye*	*Brincat et al.*	1 (case report)	Scleromalacia	Prior history of herpes zoster ophthalmicus	No treatment, follow-up, and intraocular pressure monitoring [**11**]
2006 *Ocular Immunology and Inflammation*	*Gungor et al.*	1 (case report)	Necrotizing scleritis	Based on typical skin lesions	Topical and systemic antiviral, topical corticosteroids [**12**]
2009 *American Journal of Ophthalmology*	*Pooja et al.*	9 cases	Diffuse anterior scleritis	scleroconjunctival biopsy	Oral and topical corticosteroids, oral methotrexate, infliximab, celecoxib, mycophenolate mofetil [**13**]
2012 *Ophthalmology*	*Gonzalez et al.*	35	28 diffuse anterior scleritis, 4 nodular scleritis, 3 necrotizing scleritis	Immunohistopathologic analyses of scleral biopsy - 16 cases, based on clinical evidence - 4 cases, positive titre - 5 cases, corneal hypoesthesia - 10 cases	Oral acyclovir [**14**]
2016 *Case Reports in Ophthalmological Medicine*	*Loureiro et al.*	1 (case report)	Nodular scleritis	PCR on Aqueous humour sample	Oral valacyclovir, oral corticosteroids and oral methotrexate [**15**]
2021 *Annals of Medicine and Surgery*	*Issiaka et al.*	1 (case report)	Nodular scleritis	Clinical features of skin lesions	Oral Acyclovir and corticosteroids [**16**]

This case report presented a case of bilateral necrotizing scleritis with scleral melt, though not previously reported in the literature, the diagnosis was made based on typical associated clinical findings of keratouveitis, based on previous clinical episodes and rapid response to antiviral therapy. Because of the straightforward diagnosis, scleral biopsy was not performed.

While the treatment for direct viral invasion is antiviral medication, the treatment for the immune reaction is corticosteroid therapy to suppress the immune reaction. For necrotizing scleritis, surgical options may be included to tackle problems of associated scleral melt. Antiviral drugs with judicial use of corticosteroids may act as a boon for the patient if used timely and in the right dosage. Hence, the significance of determining the pathological process through keen observation of signs and symptoms, cannot be underestimated. 

## Conclusion

This case report aimed to demonstrate a rare atypical presentation of bilateral herpetic necrotizing scleritis with scleral melt. While the diagnosis is straightforward in cases with typical findings, in atypical cases the diagnosis may be challenging. Associated clinical findings, history of herpes keratitis that may be recurrent, and response to antiviral drugs may give clues toward diagnosis in such atypical cases. In addition, surgical intervention should not be delayed if it seems inevitable. 

**Conflict of Interest Statement**

The authors state no conflict of interest.

**Informed Consent and Human and Animal Rights Statement**

Informed consent has been obtained from the patient included in the study.

**Authorization for the use of human subjects**

Ethical approval: The procedures followed were per the ethical standards of the Institutional Ethics Committee and with the Helsinki Declaration of 1975, as revised in 2000 and 2008.

**Acknowledgments**

None. 

**Sources of Funding**

None. 

**Disclosures**

None.
